# Salicylic acid-induced abiotic stress tolerance and underlying mechanisms in plants

**DOI:** 10.3389/fpls.2015.00462

**Published:** 2015-06-30

**Authors:** M. Iqbal R. Khan, Mehar Fatma, Tasir S. Per, Naser A. Anjum, Nafees A. Khan

**Affiliations:** ^1^Department of Botany, Aligarh Muslim UniversityAligarh, India; ^2^Centre for Environmental and Marine Studies, Department of Chemistry, University of AveiroAveiro, Portugal

**Keywords:** abiotic stress, crop-loss, phytohormones, salicylic acid, stress tolerance mechanisms

## Abstract

Abiotic stresses (such as metals/metalloids, salinity, ozone, UV-B radiation, extreme temperatures, and drought) are among the most challenging threats to agricultural system and economic yield of crop plants. These stresses (in isolation and/or combination) induce numerous adverse effects in plants, impair biochemical/physiological and molecular processes, and eventually cause severe reductions in plant growth, development and overall productivity. Phytohormones have been recognized as a strong tool for sustainably alleviating adverse effects of abiotic stresses in crop plants. In particular, the significance of salicylic acid (SA) has been increasingly recognized in improved plant abiotic stress-tolerance via SA-mediated control of major plant-metabolic processes. However, the basic biochemical/physiological and molecular mechanisms that potentially underpin SA-induced plant-tolerance to major abiotic stresses remain least discussed. Based on recent reports, this paper: (a) overviews historical background and biosynthesis of SA under both optimal and stressful environments in plants; (b) critically appraises the role of SA in plants exposed to major abiotic stresses; (c) cross-talks potential mechanisms potentially governing SA-induced plant abiotic stress-tolerance; and finally (d) briefly highlights major aspects so far unexplored in the current context.

## Introduction

### Abiotic Stresses and Salicylic Acid as a Major Stress Impact-Mitigation Tool in Plants

Abiotic stress has now been recognized as the biggest and potential threat for agricultural productivity all over the world. Nevertheless, anthropogenic activities in the developmental era have aggravated the degradation of agricultural system and its productivity due to major abiotic stresses such as such as metals/metalloids (hereafter termed as ‘metal/s’), salinity, ozone, UV-B radiation, extreme temperatures, nutrient (deficiency and excess), and drought (Khan and Khan, 2013; Anjum et al., 2014a). In fact, the imposed abiotic stresses can potentially influence almost all physiological, biochemical, and molecular processes in plants from the early stage of seed germination to maturity, and eventually cause severe losses in the economic yield of crop plants. It has been projected that abiotic stresses may adversely affect 70% yield of staple food crops (Kaur et al., 2008; Mantri et al., 2012). In one of the estimates of U.S. Environmental Action Group, the toxicity caused by varied metals has been one of the major concerns to the agriculture system and health of more than 10 million people in eight countries (such as China, Dominican Republic, India, Kyrgyzstan, Peru, Russia, Ukraine, and Zambia; ENS, 2006). In addition, increasing salinization of arable land is expected to result in 30% land-loss by the end of 2028 and 50% by mid of the 21st century (Wang et al., 2003). Notably, the rise in CO_2_ concentration by about 500–1000 ppm by the year 2100 has been projected to cause an increase in the mean temperature by approximately 3°C (Khan et al., 2013a). Hence, together with salinization of arable land, drought, nutrients-unavailability, metal toxicity, and climate change have been expected to significantly aggravate the problem (Anjum et al., 2014a).

Plant growth regulators play important roles in the regulation of plant developmental processes and signaling networks as they are involved either directly or indirectly in a wide range of biotic and abiotic stress responses and tolerance in plants (Khan et al., 2012a,b,c; Asgher et al., 2015). Salicylic acid (SA) is a phenolic compound involved in the regulation of growth and development of plants, and their responses to biotic and abiotic stress factors (Raskin, 1992; Khan et al., 2012a,b,c, 2013b; Miura and Tada, 2014). SA is involved in the regulation of important plant physiological processes such as photosynthesis, nitrogen metabolism, proline (Pro) metabolism, production of glycinebetaine (GB), antioxidant defense system, and plant-water relations under stress conditions and thereby provides protection in plants against abiotic stresses (Khan et al., 2010, 2012a,b,c, 2013b, 2014; Nazar et al., 2011; Miura and Tada, 2014). Apart from its involvement in the induction of defense-related genes and stress resistance in biotic stressed plants (Kumar, 2014), SA has been shown to improve plant tolerance to major abiotic stresses such as metal (Zhang et al., 2015), salinity (Khan et al., 2014; Nazar et al., 2015), osmotic (Alavi et al., 2014), drought (Fayez and Bazaid, 2014), and heat stress (Khan et al., 2013b). Exogenously sourced SA to stressed plants, either through seed soaking, adding to the nutrient solution, irrigating, or spraying was reported to induce major abiotic stress tolerance-mechanisms (Horváth et al., 2007; Khan et al., 2012a,b,c, 2013b, 2014; Anwar et al., 2013; Palma et al., 2013). SA influences plant functions in a dose dependent manner, where induced or inhibited plant functions can be possible with low and high SA concentrations, respectively. For example, in *Matricaria chamomilla*, 50 and 250 μM SA concentrations were reported to, respectively, promote and inhibit growth (Kováčik et al., 2009). In another instance, 0.1 and 0.5 mM SA promoted photosynthesis and growth of *Vigna radiata* but an inhibited growth was evidenced with 1.0 mM SA (Nazar et al., 2011). Besides the concentration of SA, the duration of the treatment, plant species, age, and treated plant organ can also influence the SA-effects in plants (Shi et al., 2009; Miura and Tada, 2014). Recent molecular studies have established that SA can regulate many aspects in plants at gene level, and thereby can improve plant-abiotic stress tolerance. SA was reported to induce several genes responsible for encoding chaperone, heat shock proteins (HSPs), antioxidants, and secondary metabolites [sinapyl alcohol dehydrogenase (SAD), cinnamyl alcohol dehydrogenase (CAD), and cytochrome P450; Jumali et al., 2011]. Additionally, SA-involvement in mitogen-activated protein kinase (MAPK) regulation, and in the expression and activation of *non-expressor of pathogenesis-related genes 1* (*NPR1*) has been evidenced (Chai et al., 2014). Nevertheless, the transcriptional reprograming that occurs during the plant defense response against biotic and abiotic stress was reported to be modulated by SA, where the transcription of different sets of defense genes can be controlled in a spatio-temporal manner via SA-mediated mechanisms (Herrera-Vásquez et al., 2015).

In order to develop ideotypes for sustainable agriculture as well as to improve overall plant performance in the conditions of the changing climate and the increased severity of abiotic stresses, it would be imperative to exploit the information available on the involvement of SA in abiotic stress tolerance in plants. In the current effort, in addition to overviewing historical background and biosynthesis of SA in plants under both optimal and stressful environments, the role of SA in plants exposed to major abiotic stress conditions is critically discussed, potential mechanisms controlling SA-induced plant abiotic stress-tolerance are cross-talked, and major aspects so far unexplored in the current context are briefly highlighted.

## Salicylic Acid: Historical Background and Biosynthesis

Centuries ago Americans, Indians, and Greeks used willow tree bark and leaves to cure aches and fevers, but it has been documented that Hippocrates prescribed the compound to relieve pain for women during child birth and fever was later recognized as SA. Ancient texts also indicate that Babylonians, Assyrians, and Chinese (Sharp, 1915) used willow (*Salix* sp.) bark and leaves for medicinal purposes. The importance of willow (*Salix* sp.) bark has also been shown in 1763 when the Reverend Edward Stone informed the Royal Society that it contained substances that relieved the symptoms of “ague” (probably malarial fever) effectively. Until 19th century the active principle of willow was not known, but later on the salicylates, methyl salicylate (MeSA), saligenin (alcohol of SA), and their glycosides were isolated from extracts of willow and other plants. The SA was isolated and purified from willow and meadowsweet in the first half of the 19th century (Leroux, 1830) and was chemically synthesized by the carboxylation of sodium phenoxide in 1860 (Kolbe and Lautemann, 1860). In 1928, Johann Buchner, a German scientist for the first time isolated SA from willow bark under the name “salicin” (the glucoside of salicyl alcohol), which was the major salicylate in willow bark (Weissmann, 1991). The name SA (Cleland and Ajami, 1974) is originated from the Latin word Salix and was given to the active ingredient of willow (*Salix* sp.) bark by Raffaele Piria in 1838. The first commercial production of synthetic SA began in Germany in 1874. Aspirin, a trade name for acetylsalicylic acid, was introduced by the Bayer Company in 1898 and rapidly became one of world’s best-selling drugs and it replaced the use of SA by producing less gastrointestinal irritation yet has similar medicinal properties. With the further advancement in scientific research in 20th century, uses of SA as a treatment for acne, psoriasis, warts, and calluses became common. The use of SA as a skin softener, for removal of dead skin cells, dirt, oil and debris and cleaning of pores is widespread. In spite of the fact that the mode of medicinal action of salicylates is a subject of continual debate, they are being used to treat human diseases ranging from the common cold to heart attacks. As aspirin undergoes spontaneous hydrolysis to SA, the two compounds have similar effects in plants.

**FIGURE 1:**
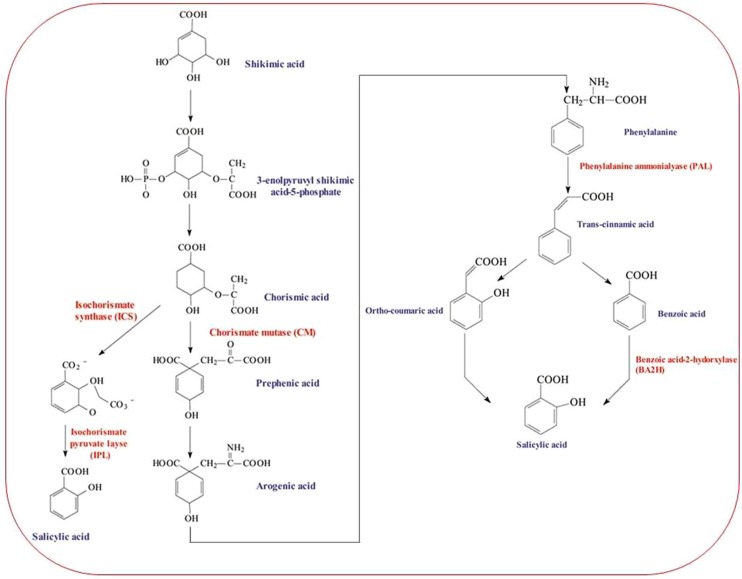
**A model of salicylic acid (SA) biosynthesis pathway starting from Shikmic acid and accomplished by three different pathways**.

Salicylic acid is a seven carbon (C) containing, naturally occurring phenolic compound and endogenously synthesized signaling molecule in plants. The shikimic acid pathway and the malonic acid pathway are the two main pathways known to be involved in the synthesis of plant phenolics. The shikimic acid pathway takes part in the biosynthesis of most plant phenolic compounds. It simply converts simple carbohydrate precursors derived from glycolysis and pentose phosphate pathway to the aromatic amino acids including SA precursor, phenylalanine (Herrmann and Weaver, 1999). The most common pathway in plants for SA synthesis is phenylalanine pathway; however, SA biosynthesis may also be accomplished by isochorismate pathway (Kawano et al., 2004; Mustafa et al., 2009). SA is produced after a series of chemical reactions catalyzed by many enzymes. The hydroxylation of benzoic acid catalyzed by enzyme benzoic acid 2-hydroxylase synthesizes SA. Benzoic acid is synthesized by cinnamic acid either via β-oxidation of fatty acids or a non-oxidative pathway (Verberne et al., 1999; Mustafa et al., 2009). Cinnamate 4-hydroxylase (C4H) catalyzes the second step in the pathway during the conversion of cinnamic acid to coumaric acid. Cinnamic acid is produced from phenylalanine by the action of enzyme phenylalanine ammonialyase (PAL). Cinnamic acid is hydroxylated to form coumaric acid followed by oxidation of the side chain, and further hydroxylated and form SA. In the third pathway, SA biosynthesis in plants has been reported from shikimic acid via chorismic acid and comaric acid (Horváth et al., 2007; An and Mou, 2011; **Figure 1**).

Multiple abiotic stress factors have been evidenced to modulate major enzymes involved in plant-SA biosynthesis. Overproduction of SA via enhanced activity of SA biosynthetic pathway enzymes (mainly isochorismate synthase, ICS; PAL) in plants helps in their protection against environmental stresses. These enzymes are key regulators of SA functions and are known to be modulated by different abiotic and biotic stress factors. In *Arabidopsis*, ICS has been found to be involved in the biosynthesis of SA during plant defense process (Wildermuth et al., 2001). Synthesis of SA following exposure to ozone in *Arabidopsis* was also suggested to involve ICS (Ogawa et al., 2005). SA application can positively regulate *ICS1* and improve drought tolerance in plants such as *Arabidopsis thaliana* (Hunter et al., 2013). In addition, mutant study on *Arabidopsis* (*ics1* and *ics2*) confirmed that in the absence of *ICS1*, *ICS2* can encode a functional ICS enzyme but in limited amounts (Garcion et al., 2008). Under salt stress, high expression of ICS and C4H enzymes can be correlated to the level of applied SA concentration. To this end, with 1.0 mM SA, induction of ICS and C4H was higher than that observed with 0.1 mM in *Carthamus tinctorius*. The isolated genes of ICS and C4H in *C. tinctorius* were considered significant for both salt stress tolerance and pathogen resistance (Dehghan et al., 2014). The involvement of enhanced PAL activity in heat and chilling tolerance was documented in *Musa* plants (Chen et al., 2008). Both Cd and Pb were reported to induce mRNA coding for PAL in *Glycine max* (Pawlak-Sprada et al., 2011). Isolation and profiling of PAL and CHS in salinity exposed *C. tinctorius* plants suggested that these two enzymes were highly responsive to 1.0 mM SA (vs. 0.1 mM SA; Dehghan et al., 2014). Recently, both water deficit and UV-B radiation were reported to cause SA accumulation as a result of increased activity of PAL and benzoic acid hydroxylase (BA2H; Bandurska and Cieslak, 2013).

## Salicylic Acid and Abiotic Stress-Tolerance in Plants

### Metals/Metalloids

Metal/metalloid-accrued stress has become a subject of great concern to sustainable agriculture and environmental system. Agricultural soils can receive metal pollutants from multiple pathways including industrial eﬄuents, burning liquid and solid fuel, urban run-off, sewage waste disposal, agricultural toxic chemicals run-off, domestic garbage dump etc. Literature is full on the significance of SA in the minimization of metal-accrued stress-impacts. Exogenously applied SA was reported to improve growth and photosynthetic traits in several crop plants including lead [Pb-(0.05, 0.15, 0.25 mmol L^-1^)] exposed *Oryza sativa* (Chen et al., 2007), Cd (10, 15, and 25 μM)-exposed *Zea mays* (Krantev et al., 2008), and Cu (0.05, 0.10, 0.15, and 0.20 mM)-exposed *Phaseolus vulgaris* (Zengin, 2014). Applied SA was evidenced to modulate antioxidant system-components and significantly decrease membrane lipid peroxidation in to Cu-exposed *P. vulgaris* (Zengin, 2014) and Pb-exposed *O. sativa* (Chen et al., 2007). Recent evidences also suggested that SA is an important regulator of photosynthesis, photosystem II (PSII), photosynthetic pigments, and the activity of enzymes such as Rubisco and carbonic anhydrase under metal stress (Al-Whaibi et al., 2012; Noriega et al., 2012; Zhang et al., 2015). Recently, an increased tolerance of *Linum usitatissimum* to Cd was attributed to SA-mediated control of H_2_O_2_ accumulation (Belkadhi et al., 2014). Ni-impact-mitigation role of SA can differ in plants exhibiting a differential Ni-accumulation. To this end, SA-biosynthetic pathway compounds and its derivative metabolites (phenylalanine, cinnamic acid, salicyloyl-glucose, and catechol) were elevated in the hyperaccumulator *Thlaspi goesingense* compared to the non-accumulators *A. thaliana* and *T. arvense* (Freeman et al., 2005). Further, the presence of SA enhanced the activity of sulfur (S) assimilation pathway enzyme (serine acetyltransferase; SAT), glutathione (GSH) content and eventually increased Ni-resistance. Exogenous SA (3.0 mM) enhanced *OsWRKY45* gene expression and increased endogenous content of SA. It has been shown that increased endogenous SA level prevented membrane damage by lowering H_2_O_2_ content in Cd-exposed *O. sativa* (Chao et al., 2010). Involvement of SA (0.5 mM) in the phytochelatins (PCs)-mediated protection of *Z. mays* was evidenced against Cd-toxicity (Szalai et al., 2013). SA can also significantly inhibit Fe-deficiency-caused chlorosis in plants (Kong et al., 2014).

### Salinity Stress

It has been estimated that about 45 million hectares of irrigated land have been damaged by salinity stress worldwide and considerable area of land affected by salinity is increasing day by day worldwide (Pitman and Lauchli, 2002; Munns and Tester, 2008). In fact, the loss of plant productivity due to salinity stress is a consequence of imbalance in cellular ionic and osmotic balances (Khan et al., 2012b). Major adverse effects of salinity stress include increased ion toxicity, osmotic stress, and nutrient-acquisition and homeostasis/deficiency, impaired stomatal conductance, increased cell-turgor loss, decreased reduction in leaf water potential, altered physiological/biochemical processes, and elevated ROS-caused oxidative stress (Munns and Tester, 2008; Nazar et al., 2011; Khan et al., 2014). The role of SA in strengthening salinity stress-tolerance mechanisms has been extensively evidenced in many crops including *Vicia faba* (Azooz, 2009), *Brassica juncea* (Nazar et al., 2011, 2015), *Medicago sativa* (Palma et al., 2013), and *V. radiata* (Khan et al., 2014).

Salicylic acid was reported to induce salinity tolerance and increased biomass of *Torreya grandis* as a result of enhanced chlorophyll content and the activity of antioxidant enzymes that eventually activated the photosynthetic process and alleviated oxidative stress (Li et al., 2014). SA-deficiency in plants was considered as a major reason of salinity-accrued increased damages and diminished activity of antioxidant enzymes in SA-deficient *NahG* transgenic of *Arabidopsis* lines (Cao et al., 2009). NaCl-accrued oxidative stress in *Hordeum vulgare* was minimized by SA (50 μM)-mediated decrease in cellular malondialdehyde (MDA, a marker of membrane-lipid peroxidation) and ROS (such as H_2_O_2_; Fayez and Bazaid, 2014; Khan et al., 2014). SA-priming can be an important strategy for enhancing major GSH-based H_2_O_2_-metabolizing enzyme such as GST. To this end, SA mitigated salinity stress-injury in *Solanum lycopersicum* by causing characteristic changes in the expression pattern of GST-gene family members such as *SlGSTT2, SlGSTT3, SlGSTF4* (Csiszár et al., 2014). Exogenously sourced SA (0.5 mM) was reported to improve salt tolerance in *Triticum aestivum* due to an enhanced transcript level of antioxidant genes; *GPX1*, *GPX2*, *DHAR*, *GR*, *GST1*, *GST2*, *MDHAR*, and *GS*, and an increased activity of ascorbate (AsA)-GSH pathway enzymes (Li et al., 2013). In another instance, SA-mediated restoration of membrane potential and prevention of salt-induced K^+^-loss via a GORK channel, and eventually improved salinity-tolerance were evinced in *A. thaliana* (Jayakannan et al., 2013).

### Ozone Stress

Ozone is among the major components of photochemical air pollution responsible for causing significant damage to both cultivated plants and forest trees (Wang et al., 2007). Ozone enters to mesophyll cells via stomata where it immediately interacts with water and other cellular components to generate phytotoxicity mainly by elevating the generation of ROS (such as $O_{2}^{\cdot -}$, H_2_O_2_,.OH, and ^1^O_2_), and triggering a series of signaling cascades and plant defense responses (Long and Naidu, 2002; Ashmore, 2005). Extensive reports are available on the key regulatory roles of SA in plant-ozone stress tolerances (Yalpani et al., 1994; Sharma et al., 1996; Yoshida et al., 2009; Khan et al., 2012a; Pál et al., 2014). SA can work as a signal molecule and promote molecular and physiological changes in ozone-exposed plants (Tamaoki, 2008). Moreover, SA is required to potentiate the antioxidant response, maintain cellular redox state, and activate processes against hypersensitive cell death and ozone-sensitivity (Rao and Davis, 1999). SA was reported to maintain plant growth, development and cellular redox system by activating the GSH-biosynthetic pathway (Yoshida et al., 2009).

In ozone-exposed *A. thaliana*, SA was involved in the accumulation of defense-related transcripts and induced resistance (Sharma et al., 1996). SA is also involved in signaling network integrating other phytohormones such as JA and/or ethylene in ozone-exposed plants (Rao et al., 2002). Notably, both SA and ethylene were evidenced by these authors to act in concert to regulate ozone-induced cell death in *A. thaliana*. In the same plant, ozone-mediated induced biosynthesis of JA or methyl jasmonate was reported to attenuate SA-dependent lesion-initiation that eventually was considered as a major factor for the decreased lesions caused by ozone (Rao et al., 2000). It has also been evidenced that ozone-accrued SA-accumulation can be promoted by ethylene-mediated regulation of the expression of the PAL and chorismate mutase (CM) genes in ozone-exposed *Nicotiana tabacum* (Ogawa et al., 2005). In another study, ozone-sensitivity in hybrid poplar was correlated with insensitivity to both SA and jasmonic acid, where these phytohormones were associated with the programmed cell death in lesion formation (Koch et al., 2000).

### UV-B Radiation

The effects of increased UV-B radiation (280–320 nm) on plant growth and development raised concerns on the need of protection mechanisms (Caldwell et al., 2007; Gill et al., 2015). Increased UV-B level can significantly diminish crop productivity by inhibiting PSII, electron transport systems, photosynthetic rate, photosynthetic pigments, nucleic acids, and biomass accumulation and partitioning (Sullivan and Teramura, 1989; Mohammed and Tarpley, 2009a,b, 2011). Exposure to UV-B radiation can decrease the expression and synthesis of key photosynthetic proteins such as chlorophyll a/b-binding proteins (Lhcb) and the D1 polypeptide of PSII (*psbA*; Jordan, 1996; Jordan et al., 1998). Significant induction in SA-accumulation was evidenced in UV radiation-exposed plants (Fodor et al., 1997; Horváth et al., 2002).

Exogenously applied SA was reported to modulate antioxidant levels, detoxify superoxide radicals, prevent oxidative damage, and protect membranes and important metabolic enzymes (Mohammed and Tarpley, 2009a). SA-mediated activation of antioxidant enzymes was considered as a major factor for SA-mediated regulation of UV-induced oxidative stress in *Capsicum annuum* leaves (Mahdavian et al., 2007). Increased photosynthetic rate, pollen viability, leaf phenolic concentration and yield in UV-B stressed-*O. sativa* was also reported (Mohammed and Tarpley, 2013). SA can decrease UV-B caused chromosome aberration level in the meristematic root tip cells (Ranceliene and Vyšniauskienė, 2012). Exogenously applied SA was reported to significantly improve photosynthetic function and its related variables in UB-B exposed plants (Karioti et al., 2008; Li et al., 2014). Earlier, UV-C radiation was also reported to upregulate the transcription of the *SA induction deficient 2* gene coding for the SA biosynthetic isochorismate synthase 1 enzyme (Martinez et al., 2004). Additionally, SA-dependent pathway was reported to control the up-regulation of the pathogenesis-related (PR) proteins (*PR-1*, *PR-2*, and *PR-5*) in UV-B-exposed transgenic *NahG A. thaliana* plants (Surplus et al., 1998).

### Temperature Stress

In the current changing environmental scenario, both low (cold and chilling stress) and high (heat) temperatures have become a potential abiotic stress-threat to crop plants. Temperature stress affects many plant-physiological and biochemical processes and induces molecular mechanisms and gene expression to modulate plants responses (Larkindale and Knight, 2002; Khan et al., 2013a,b; Kazemi-Shahandashti et al., 2014; Siboza et al., 2014). SA-supplementation has been reported to differentially benefit several plant species exposed to low/chilling temperatures (Janda et al., 1999; Ding et al., 2002; Horváth et al., 2002; Kang et al., 2012; Kazemi-Shahandashti et al., 2014; Siboza et al., 2014) and high (He et al., 2002; Larkindale and Knight, 2002; Clarke et al., 2004; Shi et al., 2006; Wang and Li, 2006; Wang et al., 2010; Khan et al., 2013a,b). SA (0.5 mM) modulated antioxidant enzymes (such as ascorbate peroxidase, APX; superoxide dismutase, SOD; guaiacol peroxidase, GPOX; GSH reductase, GR) and improved chlorophyll fluorescence in *Z. mays* under low (2°C) temperature stress (Janda et al., 1999). Exogenously SA can inhibit the activity of isozymes (CAT-1 and CAT-2) of catalase (CAT) which in turn can mediate responses of *Z. may*s to low temperature stress (Horváth et al., 2002).

Salicylic acid (2.0 mM)-mediated increased synthesis of total phenolics and the activity of PAL were reported to improve chilling tolerance in cold-stored lemon fruit (*Citrus limon*; Siboza et al., 2014). Mutlu et al. (2013) reported that exogenously sourced SA results in cold tolerance by enhancing antioxidant enzymes, ice nucleation activity, and the patterns of apoplastic proteins in *H. vulgare* genotypes. In another report, SA significantly protected ultra-structures in *Musa acuminata* seedlings under chilling stress (Kang et al., 2012). Least reports are available in literature on the molecular mechanisms underlying SA-mediated improved plant tolerance to cold/chilling temperature. Increased chilling tolerance was evidenced in chilling-exposed *S. lycopersicum* fruit as a result of low concentrations of (0.01 mM) MeSA-mediated induction in the synthesis of some stress proteins, such as PR proteins (Ding et al., 2002). The applied MeSA significantly increased accumulation of *PR-2b* and *PR-3a* mRNAs but slightly increased *PR-3b* mRNA accumulation (Ding et al., 2002).

Salicylic acid-mediated improved plant tolerance to heat stress has also been reported (He et al., 2002; Larkindale et al., 2005; Wang et al., 2010; Khan et al., 2013a,b). Larkindale and Knight (2002) reported that the transgenic *Arabidopsis* seedlings showing a bacterial SA-decomposing salicylate hydroxylase were less tolerant to heat stress. SA signaling played an important role in the acquisition of *Ocimum basilicum* to heat (Clarke et al., 2004). SA application increased antioxidant system in *Vitis vinifera* and induced activity of APX, GR, and monodehydro ascorbate (MDHA), increased redox ratios of AsA and GSH, and maintained Ca^2+^-homeostasis were reported in SA-supplemented and cold/heat treated *V. vinifera* (Wang and Li, 2006). SA (1.0 mM) decreased electrolyte leakage and oxidative stress, and improved maximum yield of PSII, Fv/Fm, and the quantum yield of the PSII electron transport in *Cucumis sativa* seedlings after both heat stress and recovery (Shi et al., 2006). Recently, Khan et al. (2013b) have shown that treatment of 0.5 mM SA can alleviate heat stress in *T. aestivum* by increasing Pro-production and restriction of the stress ethylene formation under heat stress. Notably, the details of the mechanisms of heat stress mitigation with the application of SA are not available and the area could be an open challenge at both physiological and molecular levels.

### Drought Stress

Drought has been considered as one of the most acute abiotic stresses presently affecting agriculture. Drought stress can significantly reduce photosynthesis and stomatal conductance, inhibit photosynthetic pigments synthesis and ultimately lead to reduction in growth of plants (reviewed by Hasanuzzaman et al., 2013). SA (500 μM)-supplementation to drought stressed *H. vulgare* resulted in increased net CO_2_ assimilation rate due to increased stomatal conductance and eventually in increased plant dry mass (Habibi, 2012). Exogenously applied SA can modulate important enzymatic (including monodehydroascorbate reductase, MDHAR; dehydroascorbate reductase, DHAR; GR; GSH peroxidase, GPX) and non-enzymatic (including GSH) components of AsA–GSH pathway, and also glyoxalase system (Gly I and Gly II) and decrease oxidative stress in drought-exposed plants (Alam et al., 2013). Foliar application of SA (1.0 μM) strengthened antioxidant defense system in drought-tolerant *Z. mays* cultivar to a great extent (vs. drought-sensitive cultivar; Saruhan et al., 2012). Low membrane lipid peroxidation but increased plant height and dry mass, and less wilting of leaves were reported in drought-exposed and SA (0.5 mM)-supplemented *T. aestivum* Kang et al. (2012). Recently, SA-biosynthetic enzymes (such as CS and ICS) were not correlated with the SA level, but ortho-hydroxy-cinnamic (oHCA) was correlated with SA biosynthesis and played crucial role in drought tolerance in *O. sativa* (Pál et al., 2014).

The application of 5.0 μM SA induced the expression of genes in Mitragyna speciosa (Jumali et al., 2011). Among the analyzed 292 expressed sequence tags (ESTs) randomly, the most of the genes that encode chaperone, HSPs, antioxidants and secondary metabolite biosynthesis, such as SAD, CAD, and Cytochrome P450 (CYP) responded to SA treatment. The authors correlated the gene-expression-responses to SA with signaling pathway in plants under drought stress condition. SA (0.5 mM)-mediated significantly improved growth in drought-exposed T. aestivum seedlings was argued as a result of SA-mediated enhancements in the transcription of GST1, GST2, GR, MDHAR genes (Kang et al., 2013). SA-accumulating mutants (cpr5 and acd6) exhibited stomatal closure and improved drought tolerance in A. thaliana by SA-mediated induced expression of PR genes (PR1, PR2, and PR5; Liu et al., 2013). SIZ1-mediated endogenous SAaccumulation was reported to play an important role in stomatal closure and drought tolerance in A. thaliana (Miura et al., 2013). Potential involvement of SA in the 76 identified proteins was reported in drought-exposed T. aestivum (Kang et al., 2012). Theses identified proteins were advocated to perform major physiological functions such as photosynthesis, carbohydrate metabolism, protein metabolism, stress and defense, energy production, signal transduction, and toxin metabolism. Some of the recent studies have shown that SA played significant role at different concentration by regulating many metabolic mechanisms (**Table 1**).

**Table 1:** Summary of representative studies on salicylic acid (SA)-mediated control of major abiotic stress-impacts in plants.

Plant name	Applied SA concentration	Parameters studied	Response	Reference
**Salt stress**
*Vigina radiata*	0.5 mM	Glycinebetaine (GB) production, net photosynthesis, plant dry mass	+	Khan et al. (2014)
*Torreya grandis*	0.5 mM	Chlorophyll content, net CO_2_ assimilation rates, proline content	+	Li et al. (2014)
*Glycine max*	0.5 mM	Na^+^/K^+^ ratio	-	Ardebili et al. (2014)
*G. max*	0.5 mM	Superoxide dismutase activity, ascorbate content	+	Ardebili et al. (2014)
*Hordeum vulgare*	0.05 mM	MDA content, Na^+^/K^+^ ratio	-	Fayez and Bazaid (2014)
	10^-4^ mM	Content and activity of Rubisco, Rubisco activase	+	Lee et al. (2014)
**Cadmium stress**
*Cucumis melo*	0.1 mM	Photosynthetic capacity, PSII photochemistry efficiency, water use efficiency	+	Zhang et al. (2015)
*Brassica juncea*	1.0 mM	Mineral nutrients content	+	Ahmad et al. (2011)
*G. max*	120 mM	Superoxide dismutase activity, chlorophyll, GSH content, heme-oxygenase-1 activity, relative protein amount	+	Noriega et al. (2012)
*Poa pratensis*	0.5 mM	Nutrient elements content (K, Ca, Mg, and Fe)	+	Guo et al. (2013)
*P. pratensis*	0.5 mM	Cd uptake	-	Guo et al. (2013)
*Ricinus Communis*	0.5 mM	Gas exchange, chlorophyll content	-	Liu et al. (2011)
**Nickel stress**
*Catharanthus roseus*	10^-5^ M	Content of alkaloids vincristine and vinblastine	+	Idrees et al. (2013)
**Chilling stress**
*Punica granatum*	1 and 2 mM	Total phenolics	+	Mirdehghan and Ghotbi (2014)
*Citrus limon*	2 mM	Total phenolics, activity of phenylalanine ammonialyase (PAL)	+	Siboza et al. (2014)
*Musa acuminata*	0.5 mM	Ultrastructure of chloroplast of mesophyll cells, mitochondria of mesophyll cells	+	Kang et al. (2007)
**Cold stress**
*H. vulgare*	0.1 mM	Apoplastic antioxidative enzymes, ice nucleation activity, pattern of apoplastic proteins	+	Mutlu et al. (2013)
**Heat stress**
*Triticum aestivum*	0.5 mM	Proline content, glutamyl kinase activity, gas exchange, water potential	+	Khan et al. (2013b)
**Drought stress**
*Zea mays*	0.001 mM	Leaf rolling degree, water potential, dry weight	+	Saruhan et al. (2012)
*Simarouba glauca*	0.05 mM	Polyphenol, alkaloids, flavonoid content	+	Awate and Gaikwad (2014)
**UV-B stress**
*G. max*	0.5 mM	Water use efficiency, Fv/Fm	+	Li et al. (2014)
*G. max*	0.5 mM	Flavonoid content	-	Li et al. (2014)
*Oryza sativa*	12.9 g ha^-1^	PS II activity, crop grain characteristics, total phenolics	+	Mohammed and Tarpley (2013)

*+, - symbols indicate increase or decrease, respectively*.

## Potential Mechanisms Underlying SA-Mediated Plant Stress-Tolerance

In order to enlighten the potential mechanisms underlying SA-mediated improved abiotic stress tolerance in plants, this section briefly appraises recent reports available on the interaction of SA with major osmolytes, mineral nutrients, secondary metabolites, and other phytohormones, and SA-involvement in ROS-signaling and the modulation of antioxidants.

### Interaction of SA with Osmolytes

To counteract the adverse effects of abiotic stresses-induced excess ROS production, plants have developed mechanisms facilitating their adaptation to osmotic and ionic stresses. Nevertheless, to maintain osmotic balance plants have well developed protective mechanism termed as osmoregulation mediated by osmolytes such GB, Pro, soluble sugars, amines etc. These compounds do not interfere with the other plant metabolic processes and contribute to the turgor-maintenance in stressed plants (Misra and Saxena, 2009).

Glycinebetaine is considered as an effective compatible solute for osmotic adjustment and protection against osmotic stress (Munns, 2005), salt stress (Khan et al., 2014), and also to stresses caused by heat (Wang et al., 2010) and metals (Bharwana et al., 2014). The accumulation of GB in stressed plants adjusts cell osmotic balance, stabilizes membrane integrity, prevents the dissociation of polypeptides from the PSII complex, protects Rubisco activity, and also detoxifies toxic ions (Ashraf and Foolad, 2007). Notably, SA and its analog aspirin can induce GB-accumulation in the range of 0.5–2.5 mM in plants at high levels of NaCl, drought, and cold stresses (Jagendorf and Takabe, 2001). The effectiveness of both salicylate and aspirin in the induction of GB-accumulation is a part of systematic acquired resistance and could be an important part of the reason for its induction in plants under NaCl-, drought-, and cold stresses-plants. The induction of GB might have other effects as well, such as activation of protein kinase when aroused by hyperosmotic stress (Hoyos and Zhang, 2000). SA-mediated increase in GB-level can improve overall plant growth (Misra and Misra, 2012). The increase in GB content led to the increase in biomass of *Rauwolfia serpentina*. Recently, Khan et al. (2014) have shown that alleviation of salinity-inhibited photosynthesis and growth by SA involves GB in *V. radiata*. It was shown that SA (at 0.5 mM) induced GB-accumulation through increased methionine content and suppressed excess ethylene formation under salinity stress. Similar effect was observed by these authors with the application of SA-analog, 2, 6, dichloro-isonicotinic acid on GB accumulation and alleviation of salinity-induced adverse effects on photosynthesis and growth.

The accumulation of another major osmolyte Pro is one of the adaptive mechanisms that plants operate for survival especially under salinity/osmotic stress conditions. Pro detoxifies excess ROS, adjusts cellular osmotic balance, protects biological membranes, and stabilizes enzymes/proteins (Iqbal et al., 2014). Researches have well documented that SA is involved in increasing Pro metabolism under abiotic stresses (Misra and Saxena, 2009; Khan et al., 2013b). SA (at 0.5 mM) significantly induced activity of Pro biosynthesis enzymes (such as pyrroline-5-carboxylate reductase and γ-glutamyl kinase) under salinity stress along with the increased Pro content. This increase in Pro metabolism was attributed to salinity stress tolerance in *Lens esculenta* (Misra and Saxena, 2009). Misra and Misra (2012) have shown that up-regulation of Pro biosynthesis enzymes (such as pyrroline-5-carboxylate reductase and γ-glutamyl kinase) and down-regulation of Pro oxidase activity were responsible for increased Pro level. In turn, the increased Pro level was advocated to the maintenance of the cell turgor in *R. serpentina* under salinity stress. SA-treatment (0.5 mM) alleviated heat stress in in *T. aestivum* by increasing Pro-production as a result of SA-mediated increase in γ-glutamyl kinase and decrease in Pro oxidase activity (Khan et al., 2013b). Increased Pro production was argued to improve nitrogen assimilation and to alleviate the heat stress-impact on photosynthesis. In contrast, exogenous Pro-application was evidenced earlier to induce SA production (mediated by NDR1-dependent signaling pathway) and was shown to modulate calcium (Ca)-mediated oxidative burst defense response in plants (Chen et al., 2011).

Accumulation of soluble sugars and sugar alcohol mannitol has also been reported to contribute in plant stress tolerance as osmoprotectants (Murakeözy et al., 2003; Cheng et al., 2009). SA was reported to inhibit valine and sucrose uptake in a concentration-dependent manner (10–200 μM; Bourbouloux et al., 1998). Improved plant health can also be achieved with increased contents of polysaccharides and soluble sugars, respectively, with 100 μmol L^-1^ (Yuan et al., 2014), and 0.5 and 1.0 mM SA (Luo et al., 2014).

### Interaction of SA with Mineral Nutrients

Mineral nutrition is a basic requirement for proper growth and development and survival under different environmental stress conditions. Studies have shown that mineral nutrient status in plants plays a critical role in the alleviation of abiotic stress (Iqbal et al., 2011; Nazar et al., 2011, 2015). SA can significantly modulate the uptake and metabolism of important mineral elements and thereby improve growth and development in abiotic stressed plants (Alpaslan and Gunes, 2001; Gunes et al., 2007; Chen et al., 2011; Khokon et al., 2011; Wang et al., 2011; Tufail et al., 2013; Nazar et al., 2015). In addition, the protective role of SA in membrane integrity and regulation of ions including nutrients uptake has also been reported (Alpaslan and Gunes, 2001; Gunes et al., 2007).

Salicylic acid can be involved in the regulation of uptake of several plant-beneficial elements such as Mn, Ca, Cu, Fe, P, and Zn and thereby minimize oxidative stress under Pb stress (Wang et al., 2011). SA-mediated changes in photosynthesis were attributed to the nutrients content of N, P, K, and Ca in *B. juncea* cultivars differing in salt tolerance (Syeed et al., 2011). In particular, 0.5 mM SA increased photosynthesis under salt stress by decreasing cellular Na^+^ and Cl^-^ ions, and increasing the content of nutrients. Earlier, strongly inhibited Na^+^ and Cl^-^ accumulation but stimulated N, Mg, Fe, Mn, and Cu concentrations were reported in SA-supplemented and salinity stressed *Z. mays* (Gunes et al., 2007). In another work, SA (0.5 mM)-mediated maintenance of higher K^+^/Na^+^ and Ca^2+^/Na ratios was considered as a major factor underlying SA-assisted improved growth, gas exchange, yield, and salinity tolerance in *Z. mays* (Tufail et al., 2013). Recently, Nazar et al. (2015) demonstrated that SA can improve salinity tolerance in *B. juncea* by upregulating the assimilation of S. Exogenously sourced SA (seed-soaked or soil-incorporated) can improve overall plant growth under B-toxicity and salinity stress by stimulating the accumulation of mineral elements including K, Mg, Mn, N, and P (Gunes et al., 2005).

Calcium is one of the important nutrient elements known for various structural roles under both optimal and stressful conditions in plants (White and Broadley, 2003). Literature supports a close link between SA and Ca (and Ca-signaling) in stressed plants (Liu and Zhu, 1997; Kawano et al., 1998; Chen et al., 2001, 2011; Yang and Poovaiah, 2002; Du et al., 2009). Interaction between SA and Ca^2+^ signaling might be involved in defense mechanisms induced by stress and maintenance of K^+^/Na^-^ ion selectivity (Liu and Zhu, 1997; Chen et al., 2001). SA and Ca (alone or in combination) can markedly improve salinity tolerance in plants via increasing Pro level (Al-Whaibi et al., 2012). Involvement of Pro in the induction of Ca-mediated oxidative burst and SA signaling has also been reported (Chen et al., 2011). A range of abiotic/environmental stresses (such as high temperature, UV-B stress, or salt stress) can induce the expression of Genes encoding calmodulin-binding protein (Ca-containing protein). On the other hand, SA was reported to induce calmodulin-binding proteins in abiotic stressed *A. thaliana* (Yang and Poovaiah, 2002). Ca-dependent protein kinases (CDPKs) have been shown to be involved in abiotic stress responses and may also be induced by SA (Chung et al., 2004; Leclercq et al., 2005). Kawano et al. (1998) reported that SA-induced a rapid and transient generation of superoxide anion followed by a transient increase in cytosolic free Ca ion concentration in *N. tabacum.* Khokon et al. (2011) reported that SA failed to induce Ca-Cyt oscillations in guard cells; whereas, K^+^ channel activity was suppressed by SA and lead to the stomatal closure.

### Involvement of SA in ROS-Signaling and the Modulation of Antioxidants

The generation and scavenging of varied ROS such as $O_{2}^{\cdot -}$, H_2_O_2_, and ^•^OH are usual in the normal aerobic metabolism in plants. Notably, important signal transducing roles and triggering and/or orchestrating plant responses to varied stress factors can be possible with the minimal levels of ROS. However, (abiotic) stresses cause imbalance between generation and scavenging of ROS, and eventually lead to a physiological condition known as oxidative stress. Thus, a number of consequences such as oxidative modification of vital biomolecules, cell death, and the arrest of plant growth and development can be possible under uncontrolled oxidative stress (Gill and Tuteja, 2010; Anjum et al., 2012).

Apoplastic-ROS have been found as regulators of cell death through their interplay with several other signaling pathways including SA-mediated signaling pathways (Overmyer et al., 2003). Both endogenous and exogenous SA was evidenced to play roles in antioxidant metabolism and have a tight control over cellular ROS (Kang et al., 2014; Khan et al., 2014). However, the coordination of SA-dependent and independent signaling components with ROS-signaling provided an appropriate defense response. SA can act as a signal for the development of the systemic acquired resistance (Shirasu et al., 1997), and can also induce the activation of a protein kinase (Mikolajczyk et al., 2000). SA was evidenced as a major signaling molecule required for agonizing ozone-induced GSH-based defense gene expression in *A. thaliana* (Rao and Davis, 1999). *A. thaliana* plants were able to recognize the response of ROS–SA interaction via an antagonistic action of SA and SA-signaling on apoplastic ROS-signaling (Xu and Brosché, 2014). SA-signaling except from the well-documented role in the regulation of defense responses was also evidenced to be involved in the regulation of light-acclimation processes and thereby influencing photosynthetic function (Mullineaux and Karpinski, 2002; Gawroński et al., 2013).

Involvement of SA in the modulation of antioxidant metabolism has been widely reported to control plant-tolerance to major abiotic stresses including ozone, UV-B, heat, metal, and osmotic stress (Wang et al., 2010; Nazar et al., 2011; Khan et al., 2012a,b,c, 2013a,b, 2014). SA-pretreatment was evidenced to alleviate the adverse effects of salinity stress on photosynthesis and growth in *V. radiata* through enhancing the activities of antioxidant enzymes including SOD, CAT, GPX, APX, and GR (Khan et al., 2014). Activities of H_2_O_2_-metabolizing enzymes (such as CAT, POD, and APX) and superoxide-dismutating enzymes (SOD) were also modulated with SA in plants exposed to drought (Saruhan et al., 2012) and cold (Mutlu et al., 2013). SA-application (at 0.5 mM) increased activity of enzymes of AsA–GSH pathway resuled in the increased tolerance of *B. juncea* to salinity stress (Nazar et al., 2015). AsA and GSH, as redox active compounds have been extensively reported to maintain a homeostatic balance of the cellular redox state, and are involved in protective mechanisms against both abiotic and biotic stresses (Anjum et al., 2010, 2014b; Noctor et al., 2012; Khan et al., 2015a). In Cd-exposed *T. aesticum* varieties, SA-signaling was correlated with GSH-related mechanisms (Kovács et al., 2014). Exogenous SA (0.5 mM) significantly improved salinity (250 mM NaCl) tolerance in *T. aestivum* by markedly increasing the pools of AsA and GSH (Li et al., 2013).

Salicylic acid-mediated differential regulation of the transcript levels of the genes encoding AsA–GSH cycle enzymes GPX (*GPX1*), phospholipid hydroperoxide GPX (*GPX2*), and DHAR (*DHAR*), GR (*GR*), GST (*GST1* and *GST2*), MDHAR (*MDHAR*), and GSH synthetase (*GS*) was advocated as a major mechanisms underlying previous role of SA in salinity tolerance via AsA and GSH (Li et al., 2013). Recently, in *S. lycopersicum*, SA-priming exhibited a concentration-dependent modulation of GST-supergene family, where SA-mitigated the salinity stress-injury and caused characteristic changes in the expression pattern of *SlGST*s only at 10^-4^ M concentration (Csiszár et al., 2014). In particular, *SlGSTF4* displayed a significant up-regulation in the leaves, while the abundance of *SlGSTL3*, *SlGSTT2*, and *SlGSTZ2* transcripts were enhanced in the roots of plants primed with high SA-concentration. SA-mediated induction in SOD and GSH-based H_2_O_2_metabolizing enzymes namely GPX and GST was argued to improve in ozone tolerance in plants (Abarca et al., 2001; Milla et al., 2003). SA (500 μM) improved Cd-tolerance and photosynthetic capacity in hemp (*Cannabis sativa*) by enhancing both SOD and POD (Shi et al., 2009). In another instance the applied SA-induced SOD activity accompanied an increase in shoot Ca^2+^ (a second messenger) and caused a transient increase in H_2_O_2_ which in turn was argued to induce antioxidant enzymes and eventually to decrease in cellular ROS (Arfan, 2009).

### Interaction of SA with Major Secondary Metabolites

Secondary metabolites, substances biosynthetically derived from primary metabolites, produced by plants as defense chemicals and are not involved in plant metabolic activity. Examples of secondary metabolites include including terpenes, phenolics, and compounds with N (alkaloids, cyanogenic glucosides, non-protein amino acids) and S (GSH, glucosinolates, phytoalexins, thionins, defensins, and allinin; Irchhaiya et al., 2015). Secondary metabolites have also been established as beneficial for plants tolerance under biotic and abiotic stress (Ramakrishna and Ravishankar, 2011).

**FIGURE 2:**
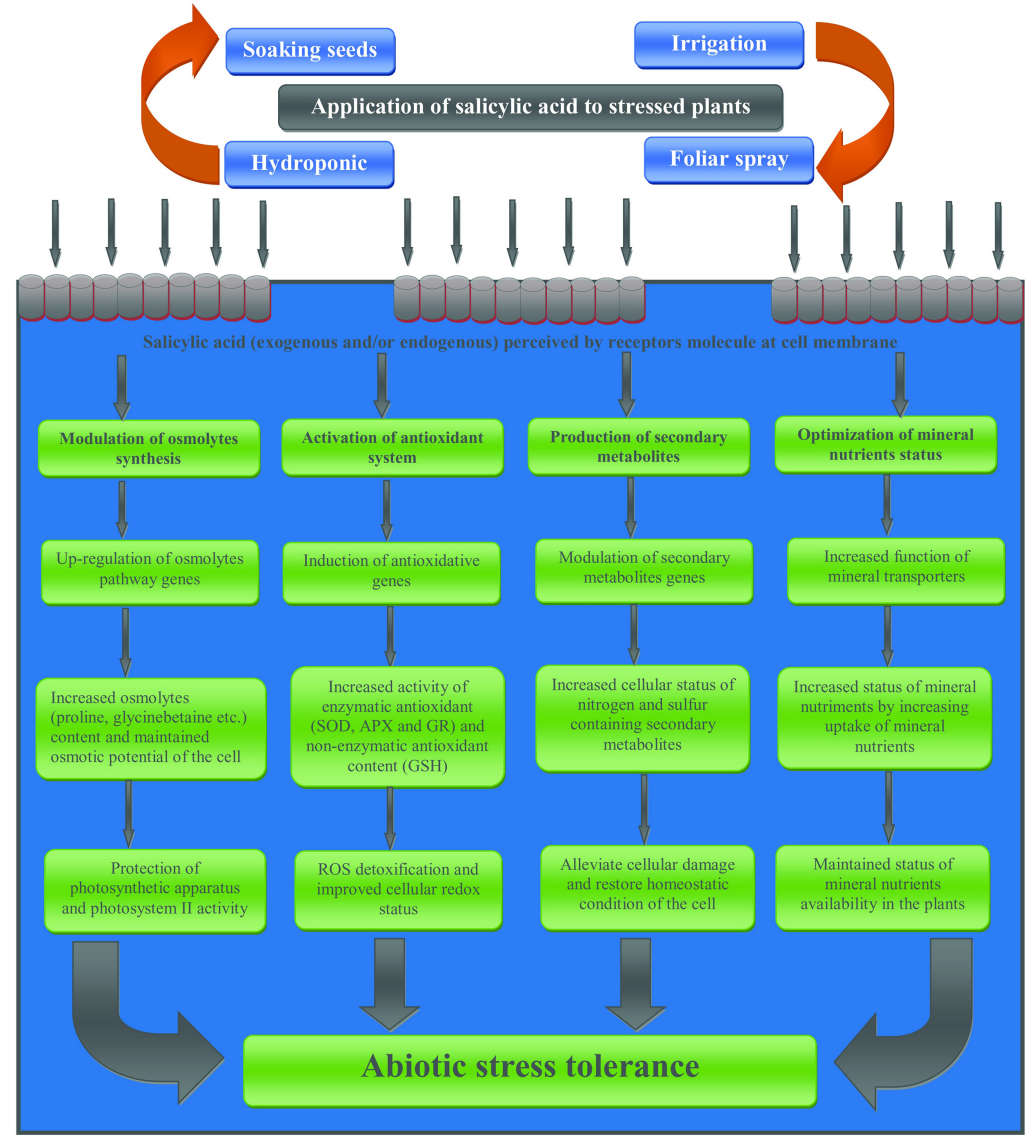
**Simplified schemes representing potential mechanisms underlying SA-mediated plant abiotic stress tolerance**.

Extensive reports are available on the direct and indirect involvement of SA in the induction of the secondary metabolite synthesis in plants (Kiddle et al., 1994; Ali et al., 2007; Idrees et al., 2013). SA-mediated induction of glucosinolates production was reported in *Brassica napus* (Kiddle et al., 1994). Exogenous application of acetyl-SA to tumor lines of *Catharanthus roseus* elevated therein the production of important secondary metabolites such as alkaloids, phenolics, furanocoumarins, and anthocyanins (Godoy-Hernández and Loyola-Vargas, 1997). Recently, in the same plant SA-mediated restoration of Ni-induced inhibition was attributed to SA-assisted improved the content of alkaloids such as vincristine and vinblastine (Idrees et al., 2013). Role of exogenously applied SA was reported in UV-B exposed *T. aestivum*, where SA increased the accumulation of anthocyanin and tocopherol, and also modulated the expression of *PR* proteins (Horváth et al., 2007). Eliciation in the PAL activity and subsequent increased vanillin production were reported in SA-supplemented in *C. chinense* (Rodas-Junco et al., 2013). Foliar application of SA at the rate of 50 mg L^-1^ induced the level of secondary metabolites such coumarins, sterols, xanthoproteins, cardiac glycosides and saponins, and ameliorated water stress in *Simarouba glauca* (Awate and Gaikwad, 2014). Earlier, SA-mediated improved thermo-tolerance in *V. vinifera* was advocated as a result of SA-induced accumulation of *PAL* mRNA, the synthesis of new PAL protein, and significant accumulation of phenolics (Wen et al., 2008). A schematic presentation of SA-mediated metabolisms responsible for abiotic stress tolerance is shown in **Figure 2**.

### Interaction of SA with Other Hormones

Many factors are involved in a complex process of plant growth and development. SA can regulate various aspects of plant responses under both stressful and optimal environments through signaling cross-talks with other phytohormones (Horváth et al., 2007; Asensi-Fabado and Munné-Bosch, 2011; Khan et al., 2012a,b, 2013b, 2014). The interaction between SA and other phytohormones such as auxin (Iglesias et al., 2011; Khan et al., 2012a), cytokinin (Peleg et al., 2011; Khan et al., 2012a), gibberellins (Alonso-Ramírez et al., 2009; Khan et al., 2012a), abscisic acid (Szepesi et al., 2009; Khan et al., 2012a), ethylene (Khan et al., 2012a, 2013b, 2014), nitric oxide (NO; Khan et al., 2012a; Kong et al., 2014), and brassinosteroids (Divi et al., 2010; Khan et al., 2012a) has been established under both normal and stressed conditions.

The outcomes of the SA-interaction with other phytohormones can be either from synergistic or antagonistic relation therein under optimal and stressful conditions. SA-mediated repression of auxin signaling was demonstrated by Iglesias et al. (2011). The authors showed that the expression of the *PR1*, a SA-induced gene in *tir1 afb2* (auxin perception small family of F-box proteins) mutant plants plays a significant role in auxin-signaling in stressed plants. Furthermore, in these mutants, *PR-1* was induced to a great extent as compared with SA-treated wild-type plants, where the negative effect of SA on auxin signaling indicates the occurrence of an antagonism between auxin and SA-signaling pathways (Iglesias et al., 2011). SA triggered the accumulation of ABA under both normal and salinity stressed plants which in turn helped in the osmotic adaptation and improved photosynthetic pigments and growth attributes in *S. lycopersicum* (Szepesi et al., 2009). ABA may alter the SA-related abiotic (cold) stress response in plants. To this end, in chilling-exposed *Z. mays*, ABA treatment induced changes in the endogenous SA and the content of oHCA, and suggested that SA related stress responses may overlap with ABA-induced cold-acclimation (Szalai et al., 2011).

The increased ethylene production under environmental stress referred as stress ethylene is believed to induce oxidative stress (Khan and Khan, 2014; Khan et al., 2015b). Reports suggest an antagonism between SA and ethylene. Under stressful conditions, the application of SA could inhibit ethylene biosynthesis by restricting the conversion of 1-aminocyclopropane carboxylic acid (ACC) to ethylene (Leslie and Romani, 1986). Exogenous SA was reported to alleviate heat stress by increasing Pro-metabolism and restricting ethylene formation in heat-stressed plants to an optimal range by inhibiting activity of ACC synthase (ACS; Khan et al., 2013b). Increased methionine and GB-accumulation in *V. radiata* plants was concomitant with improved photosynthesis and growth, and the suppression of ethylene formation as a result of SA (0.5 mM)-mediated inhibition in ACS activity under salinity stress (Khan et al., 2014). Salinity stress was evidenced to induce cell death mainly by ethylene-induced ROS-production; however, ROS generated by SA was not controlled by ethylene in *Lycopersicon esculentum* cell suspension (Poór et al., 2013). In contrast, Ghanta et al. (2014) suggested a synergistic cross-talk between ethylene and SA for combating environmental stress-impact in plants. In a similar study, an increased ethylene formation was observed with SA-addition in the MS medium; whereas, it was decreased in regenerants exposed to AgNO_3_ in *Prunus persica* rootstock (Molassiotis et al., 2005). In contrast, the accumulation of polyamines under salinity stress was prevented by SA-application along with the increased ACC content (Palma et al., 2013). Further, SA-induced inhibition in the lipid peroxidation and polyamines in nodules of *M. sativa* can be an indication of SA-mediated activation of ethylene-dependent hypersensitive response (Palma et al., 2013).

Nitric oxide, a major ubiquitous signal in plant systems, plays significant roles in a wide range of responses to environmental and endogenous cues (Freschi, 2013). SA is among the major phytohormones to which NO interacts, plays role as a second messenger, and controls stomatal movement in higher plants (Hao et al., 2010). NO has been advocated to act as downstream of SA-signaling in the reduction of induced oxidative damage in osmotic stressed *T. aestivum* seedlings (Naser Alavi et al., 2014). SA can also stimulate the synthesis of NO via enhancing the activity of NO–synthesizing enzymes (Zottini et al., 2007). Under Ni stress, addition of SA or SNP partially reduced the toxic effects of Ni. However, Ni-stressed plants supplemented with SA+NO exhibited an improved growth and photosynthetic pigments in canola (*B. napus*; vs. Ni-treated plants; Kazemi et al., 2010). Recently, the combined application of SA and NO benefitted *Arachis* seedlings (in terms of high Fe-uptake and less leaf interveinal chlorosis) maximally compared to exogenous individual SA and SNP under Fe-deficiency (Kong et al., 2014). The stress tolerance conferring ability of brassinosteroid in plants was advocated to occur in part in its interactions with other stress hormones such as SA (Divi et al., 2010). It was observed that *NPR1* gene, a master regulator of SA-mediated defense genes is a critical component of 24-epibrassinloide-mediated increase in thermo- and salinity tolerance in *A. thaliana* (Divi et al., 2010). There occur antagonistic interactions between SA and JA at the levels of MAPKs signaling and biosynthesis (Khan et al., 2012a,b). The antagonistic relationship between SA and JA can also modulate the expression of PR protein genes, where induction and inhibition of *PR* genes can be possible with SA and JA, respectively, (Thaler et al., 1999; Wang et al., 2001).

## Conclusion and Future Prospects

Abiotic stress has been recognized as a major threat to the agricultural system. To cope up the adverse effects of abiotic stress, plants induce several physiological processes and molecular mechanisms. Investigations have shown SA as a strong and potential tool in reducing or alleviating the adverse effects of abiotic stress in plants. Application of SA has been shown to be beneficial for plants either in optimal or stress environments. SA can regulate various plant metabolic processes and modulate the production of varied osmolytes and secondary metabolites, and also maintain plant-nutrient status hence, to protect plants under abiotic stress conditions. Literature appraised herein confirmed the focus of the studies related with SA in abiotic stressed plants mainly on unveiling various physiological/biochemical processes.

There are still a few of interesting questions awaiting further investigation. For example, *NPR1*, *3*, and *4* are discovered as the SA receptor in plants. However, it is required to perform exhaustive molecular studies cross-talking *NPR1*, *NPR3*, and *NPR4*, and their cumulative potential role in SA-perception in orchestrating defense gene expression in abiotic stressed plants. Unveiling intricacies of the complete SA-signaling in abiotic stresses, and the relationship between distinct facets of SA in plant immunity and abiotic stresses responses would be fascinating and rewarding. Meanwhile, more genomics and proteomics studies are expected to broadly reveal SA-responsive genes and proteins upon stresses. Since SA influences the plant functions in a dose dependent manner and a high SA-concentration doesn’t profit stress tolerance, abiotic stress-regulated SA-catabolism maybe a field worth further investigation. Molecular dissection is also required to unravel insights into the SA-mediated control of other the production and/or signaling of specific plant hormones/metabolites, and their feed-back influence in the machinery responsible for controlling SA-endogenous levels. Overall, finer details of SA-mediated defense networks/plant-immunity as well as further insights into the cross-talk of SA with other defense signaling pathways in abiotic stressed plants can be uncovered through adopting an integrated approach incorporating genetics, molecular biology, biochemistry, genomics, bioinformatics techniques, and computational biology.

## Conflict of Interest Statement

The authors declare that the research was conducted in the absence of any commercial or financial relationships that could be construed as a potential conflict of interest.
